# Dynamic Evolution Pattern of Water Distribution, Storage Stability, and Reheating Properties in Fresh Wet Tuber-Based Vermicelli: From the Perspective of Moisture Regulation Strategies

**DOI:** 10.3390/foods15030553

**Published:** 2026-02-04

**Authors:** Cui Guo, Xinkui Niu, Jiayin Zhu, Bo Liu, Siyuan Liu, Xianli Cao, Lijuan Wang

**Affiliations:** 1 Food Laboratory of Zhongyuan, Department of Nutrition and Health, China Agricultural University, No. 17 Tsinghua East Road, Haidian District, Beijing 100083, China; guocui0101@163.com (C.G.); 18339240061@163.com (X.N.); jiayin_0107@163.com (J.Z.); 19003857771@163.com (B.L.); siyuan.liu@cau.edu.cn (S.L.); 2 Luohe Yu Dingyuan Food Industry Co., Ltd., No. 66 Advanced Manufacturing Development Zone, Yancheng District, Luohe 462044, China; wuyang5688@163.com

**Keywords:** fresh wet tuber-based vermicelli, Immersion time, water distribution, texture, physicochemical properties

## Abstract

Fresh wet tuber-based vermicelli is prized for its soft and elastic texture, which relies on high moisture content. However, this leads to water exudation and texture hardening during storage, limiting industrial application. This study employed immersion as a moisture regulation strategy, analyzing changes in water distribution, hardness, microstructure, and reheating quality during immersion and storage. Results indicated that moisture content increased rapidly within the first 20 min and stabilized after 40 min, accompanied by a significant reduction in hardness and gelatinization enthalpy. Microstructural analysis revealed that an immersion time of 30–40 min (62–63% moisture) was optimal, preserving suitable hardness and structural integrity. During storage, these samples achieved stable water distribution by day 35. In contrast, samples immersed for 50–60 min (64–65% moisture) showed markedly increased free water and notable structural damage. Reheating tests further confirmed that immersion for 30–40 min helped maintain moderate hardness post-storage. Therefore, controlling immersion to 30–40 min effectively balances texture, storage stability, and reheating quality.

## 1. Introduction

Vermicelli, a traditional Asian staple food, is primarily processed from starch and water [1]. Based on their primary starch ingredients, vermicelli can be classified into bean vermicelli, tuber-based vermicelli, grain vermicelli, and other types [2,3]. Fresh wet tuber-based vermicelli has gained popularity in recent years due to its delicate and smooth texture, short cooking time, and convenient and palatable consumption [1]. Nevertheless, fresh wet tuber-based vermicelli, without undergoing drying treatment, has a high water content, which limits its storage period. During storage, it is prone to problems such as water evaporation and condensation, hardening and cracking of the texture, and accelerated starch retrogradation, significantly affecting the quality of the product.

The preparation process of fresh wet tuber-based vermicelli primarily includes thickening, dough kneading, extrusion, heat processing, cooling, and immersion [4]. The immersion process is one of the core procedures that determine the final quality of the product. Moderate immersion of fresh wet tuber-based vermicelli facilitates the formation of a dense and flexible gel network [5]. As the immersion time increases, the starch gel absorbs water, and the water content increases. This will shorten the heating time before consumption and inhibit its long-term retrogradation [6]. However, excessively long immersion time not only reduces production efficiency but also damages the starch gel structure, resulting in a decline in cooking quality [7].

Water is a key factor in determining the appearance, microstructure and physicochemical properties of food [8]. As an indispensable additive in the production of fresh wet tuber-based vermicelli, water interacts with starch and other macromolecules, providing a structural basis for the elasticity of vermicelli. Water serves as an excellent plasticizer for starch [9]. It not only affects the degree of gelatinization of starch, but also affects starch regeneration by affecting the migration rate of amylose and amylopectin chains [5]. Earlier studies found that in wheat starch systems, when the moisture content is between 27% and 50%, the retrogradation rate of the sample increases with increasing moisture content. However, when the moisture content is between 50% and 90%, the retrogradation rate decreases with increasing moisture content [10]. The study by Jane et al. mentioned that the degree of retrogradation in coix starch decreased with increasing moisture content [11]. Zhang et al., in their study on rice starch, found that under conditions of relatively high moisture content, a reduction in water levels increased the probability of molecular collisions between starch molecules, promoting cross-linking and leading to pronounced retrogradation [12]. Thus, this study aimed to investigate the impact of immersion time on the quality, storage stability, and reheating properties of fresh wet tuber-based vermicelli. Specifically, we analyzed the moisture characteristics (content, absorption rate, distribution), textural properties (hardness), microstructure, thermal properties, and their evolution during storage and after reheating of vermicelli subjected to different immersion times (0–60 min). The findings provide theoretical support for selecting immersion time and moisture content during the production process of fresh wet tuber-based vermicelli and predicting storage quality.

## 2. Materials and Methods

### 2.1. Materials

The main ingredients for preparing fresh wet tuber-based vermicelli included edible corn starch (starch content: 86.8%, moisture: 13%, ash content: 0.1%, protein content: 0.1%; Shandong, China), edible cassava starch (starch content: 87.6%, moisture: 12%, ash content: 0.2%, protein content: 0.2%; Thailand), and edible potato starch (starch content: 81.7%, moisture: 18%, ash content: 0.2%, protein content: 0.1%; Ningxia, China). Additional materials comprised soybean oil (Grade 1, refined; Hebei, China), edible salt (Refined iodized salt; Shanxi, China), food-grade polyethylene vacuum preservation bags (Zhejiang, China), and Polytetrafluoroethylene (PTFE) tape (thickness 0.1 mm; Zhejiang, China) employed for wrapping samples during moisture distribution analysis.

### 2.2. Preparation of Fresh Wet Tuber-Based Vermicelli with Different Immersion Times

The vermicelli was prepared using the following formulation (by weight): edible potato starch (6.5%), edible cassava starch (37%), edible corn starch (17.5%), edible salt (1.3%), and water (37.7%). First, the potato starch and salt were mixed with hot water at 60 °C (at a starch-to-water ratio of 1:3.4) and subjected to pre-gelatinization. Subsequently, the cassava starch, corn starch, and the remaining water (25 °C) were added. The mixture was stirred at 36 r/min for 20 min to form a dough, which was then extruded into strips using a forming machine (model YY-30, Xingtai Yunyang Machinery Factory, Xingtai, China). The strips were boiled in boiling water for 30 s and subsequently cooled in 25 °C water for 30 s. They were then subjected to gradient immersion in 25 °C water (ranging from 0 to 60 min, at 10 min intervals). After being taken out and drained, the samples were immediately subjected to testing for texture, moisture content, water distribution, thermodynamic properties, and microstructure. Samples corresponding to different immersion times (0, 10, 20, 30, 40, 50, and 60 min) were designated as B0, B10, B20, B30, B40, B50, and B60, respectively.

### 2.3. Storage of Fresh Wet Tuber-Based Vermicelli with Different Immersion Times

To investigate the effect of storage, the prepared vermicelli samples were subjected to the following storage procedure. Fresh wet tuber-based vermicelli was divided into 150 g portions and packaged in food-grade polyethylene bags (20 cm × 25 cm). The bags were sealed tightly to prevent moisture loss but were not vacuumed. The packaged samples were steamed at 95 °C for 25 min in a commercial steam cabinet (model KZ-80D, Guangdong Demashi Intelligent Kitchen Equipment Co., Ltd., Guangzhou, China), followed by natural cooling in still air until equilibrated to ambient temperature (25 ± 1 °C). Subsequently, the samples were stored under refrigeration at 4 °C for 1, 7, 21, 35, and 49 days.

### 2.4. Determination of Moisture Content

A sample of fresh wet tuber-based vermicelli (2–3 g) was accurately weighed, and its moisture content was determined following Method I (Direct Drying Method) of the Chinese National Food Safety Standard GB 5009.3-2016 [13], using an analytical balance (model FA124X, Tianjin Detent Sensing Technology Co., Ltd., Tianjin, China) and an electric blast drying oven (model DHG-9070A, Jinghong Experimental Equipment, Shanghai, China).

### 2.5. Determination of Moisture Distribution

A 1.5 g sample was accurately weighed, tightly wrapped with PTFE Tape, and placed in a test tube with an internal diameter of 25 mm. The transverse relaxation time (T_2_) was measured using a low-field nuclear magnetic resonance (LF-NMR) spectrometer (model NMI-20-060, Niumag Corporation, Suzhou, China). The instrument parameters were set as follows: sampling frequency, 200 kHz; number of scans, 8; recycle delay, 2000 ms; number of echoes, 8000. Signal acquisition was conducted using the Niumag NMR Analyzer software (model Version 4.0, Niumag Corporation, Suzhou, China). The acquired signals were subsequently processed via the built-in T_2_ inversion algorithm to obtain the relaxation time distribution.

### 2.6. Determination of Texture

A segment of fresh wet tuber-based vermicelli (5 cm to 8 cm in length) was selected and placed horizontally on the testing platform, ensuring gap-free contact with the platform and direct alignment below the detection probe. Texture profile analysis (TPA) was performed with six replicates per sample group using a texture analyzer (model CTX, AMETEK, Berwyn, PA, USA). The test parameters were set as follows: probe, P/36R cylinder; trigger force, 5 g; target deformation, 50%; pre-test speed, 2 mm/s; test speed, 1 mm/s; post-test speed, 5 mm/s. Texture analysis was performed on samples under two conditions: (1) non-reheated samples directly from storage, to directly assess the intrinsic structural hardening due to starch retrogradation; and (2) samples after the reheating treatment described in Section 2.9, to evaluate the final eating quality, which depends on the gel’s ability to withstand cooking and rehydration.

### 2.7. Determination of Thermodynamic Properties

The vermicelli samples were freeze-dried prior to thermal analysis. The freeze-drying procedure was as follows: samples were first frozen at −80 °C for 12 h, then dried in a freeze dryer (model THLG-12A, Tuohue Electromechanical Technology Co., Ltd., Shanghai, China) with the condenser temperature set at −80 °C and a vacuum pressure below 20 Pa. The samples were freeze-dried for 48 h until constant weight was achieved. An accurately weighed 3 mg sample of freeze-dried vermicelli powder was placed in an aluminum crucible. After the addition of 9 mg of deionized water, the crucible was hermetically sealed and equilibrated overnight at 4 °C. Thermal analysis was performed using a differential scanning calorimeter (model DSC4000, PerkinElmer, Waltham, MA, USA) with an empty crucible as a reference, with the following temperature program: heating from 20 °C to 120 °C at a constant rate of 10 °C/min under a nitrogen atmosphere. The gelatinization temperature range and retrogradation enthalpy (ΔH) were determined using the Netzsch Proteus Analysis software (model Version 6.0, PerkinElmer, Waltham, MA, USA).

### 2.8. Determination of Microstructure

For microstructure observation, samples were freeze-dried following the identical procedure described in Section 2.7. The lyophilized samples were then cross-sectioned, mounted on aluminum stubs with conductive tape, and sputter-coated with gold using an ion sputter coater (model MC1000, Hitachi, Tokyo, Japan). Microstructure was examined using a field-emission scanning electron microscope (model SU8010, Hitachi, Tokyo, Japan) under the following conditions: an acceleration voltage of 3 kV and a magnification of 1000×.

### 2.9. Determination of Water Absorption After Reheating

The experimental procedure was carried out by adopting the method of Huang [14] with slight modifications. A total of 50 g of fresh wet tuber-based vermicelli that had been stored for 1, 7, 21, 35, and 49 days was placed into a 1 L beaker. Subsequently, 500 mL of boiling water was added, and the vermicelli was separated by stirring. After 90 s of immersion, the fresh wet tuber-based vermicelli was taken out and cooled in tap water (25 ± 1 °C) for 15 s. The sample was then placed in a perforated spoon and drained for 60 s to remove excess surface water. The mass of hydrated vermicelli (m_1_) was recorded using an analytical balance (model FA124X, Tianjin Detent Sensing Technology Co., Ltd., Tianjin, China). The water absorption rate (WAR) was calculated using the following formula:

$$\mathrm{WAR}\;(\%)\;=\;\frac{m_{1}-50}{50}\times 100\%$$

where m_1_ is the mass of the hydrated fresh wet tuber-based vermicelli (g).

### 2.10. Statistical Analysis

All data are expressed as the mean ± standard deviation of triplicate measurements. Statistical analyses were performed using Statistical Package for the Social Sciences (SPSS) 16.0, with significant differences determined at *p* < 0.05 by one-way analysis of variance (ANOVA) and Duncan’s multiple range test. The graphical presentation was generated using Origin 2019b.

## 3. Results and Discussion

### 3.1. Effect of Immersion Time on the Physicochemical Properties of Fresh Wet Tuber-Based Vermicelli

#### 3.1.1. Water Content Analysis

**Figure 1:**
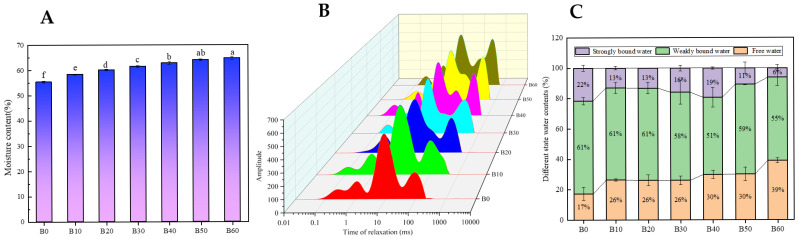
(**A**) Moisture content of fresh wet tuber-based vermicelli at different immersion times. Different letters in sub-figure A indicate statistically significant differences (*p* < 0.05). (**B**) Transverse relaxation time spectra of fresh wet tuber-based vermicelli with different immersion times. (**C**) Different state water contents of fresh wet tuber-based vermicelli with different immersion times.

The initial moisture content of fresh wet tuber-based vermicelli played a critical role in its textural changes, moisture migration, and starch retrogradation during storage. As shown in Figure 1A, the moisture content of the unimmersed samples was 55.37%, which increased significantly (*p* < 0.05) to 64.82% over the 0–60 min immersion period. A rapid increase from 55.37% to 60.17% occurred within the initial 20 min. Subsequently, the rate of moisture gain decelerated, and no statistically significant increase (*p* > 0.05) was observed beyond 40 min of immersion, indicating that moisture content had plateaued. This pattern was attributed to the significant moisture gradient between the interior of the vermicelli and the external environment during the initial stage of immersion, which drove the rapid migration of water to the interior by capillary action. As the system approached water adsorption equilibrium with prolonged immersion, the absorption rate decreased [7].

#### 3.1.2. Moisture Distribution Analysis

Figure 1B shows the transverse relaxation time (T_2_) spectra of fresh wet tuber-based vermicelli subjected to different immersion times. Within the range of 0.01–10,000 ms, three to four distinct peaks were observed. Specifically, samples B10 and B20 exhibited a singular peak for strongly bound water, whereas all other samples (B0, B30, B40, B50, B60) showed bimodal peaks. The moisture in fresh wet tuber-based vermicelli consists of three components: strongly bound water, weakly bound water, and free water. The distribution of water reflects the closeness between the starch molecular matrix and water molecules. Strongly bound water, which primarily associates with starch via hydrogen bonding, is characterized by relaxation times T_21_ and T_22_; Weakly bound water, located within the starch gel network, is represented by T_23_; Free water which adheres loosely to the gel surface, is indicated by T_24_ [5]. A larger transverse relaxation time value indicates greater water mobility, whereas a smaller value indicates weaker mobility [15].

As presented in Table 1, the values of T_23_ and T_24_ increased significantly (*p* < 0.05) with prolonged immersion time, indicating that the higher moisture content enhanced water mobility within the vermicelli. This can be attributed to the limited availability of hydrogen-bonding sites on starch molecules, which restricts the extent of water binding. As a result, increased moisture levels reduce structural constraints and promote greater molecular mobility. These findings are further corroborated by Figure 1C, which shows a significant increase (*p* < 0.05) in the percentage of free water with extended immersion time.

#### 3.1.3. Hardness Analysis

**Figure 2:**
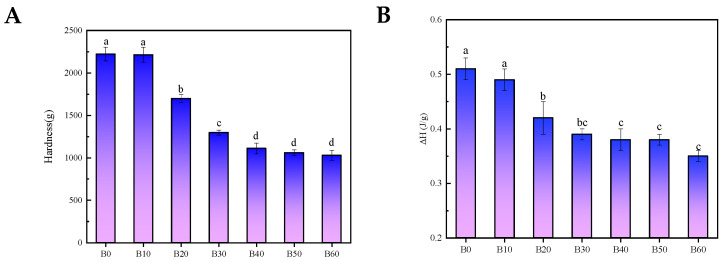
(**A**) Hardness of fresh wet tuber-based vermicelli at different immersion times. (**B**) Thermal enthalpy value of fresh wet tuber-based vermicelli at different immersion times. Different letters in the figure indicate statistically significant differences (*p* < 0.05).

As shown in Figure 2A, the hardness of fresh wet tuber-based vermicelli progressively decreased (*p* < 0.05) with increasing immersion time, declining from 2224 g for sample B0 (0 min) to 1029 g for sample B60 (60 min). Moreover, after 40 min of immersion, there were no significant differences in hardness (*p* > 0.05). This phenomenon can be attributed to enhanced water absorption during extended immersion, which promotes water penetration between starch granules. As a result, the expansion of the gel pore structure compromises the structural integrity of the vermicelli, leading to a marked reduction in hardness [16].

#### 3.1.4. Thermodynamic Properties Analysis

Starch gelatinization is an endothermic process. The absorbed heat primarily facilitates the disruption of the starch crystalline structure, the swelling of granules, and the leaching of amylose molecules from the starch matrix. A more tightly packed molecular arrangement within the starch granules requires more heat for complete gelatinization. Consequently, a higher enthalpy value observed during re-gelatinization indicates a greater extent of retrogradation [17]. As shown in Figure 2B, the enthalpy change (ΔH) of the fresh wet tuber-based vermicelli decreased significantly (*p* < 0.05) with increasing immersion time, declining from 0.51 J/g for sample B0 to 0.35 J/g for sample B60. No significant difference in ΔH was observed after 40 min of immersion (*p* > 0.05). This decrease in enthalpy is likely attributable to the increased moisture content resulting from prolonged immersion, which dilutes the starch molecules. This dilution effect diminishes the opportunity for intermolecular cross-linking and the ordered re-association of polymers, thereby reducing the extent of starch retrogradation [12] and manifesting as a lower ΔH value.

Furthermore, these findings suggest that a higher moisture content enhances the degree of gelatinization in fresh wet tuber-based vermicelli. The increased moisture enables more complete hydration and expansion of starch granules, leading to extensive disruption of hydrogen bonds, more thorough gelatinization, and a more uniform aging profile [18].

#### 3.1.5. Microstructure Analysis

**Figure 3:**
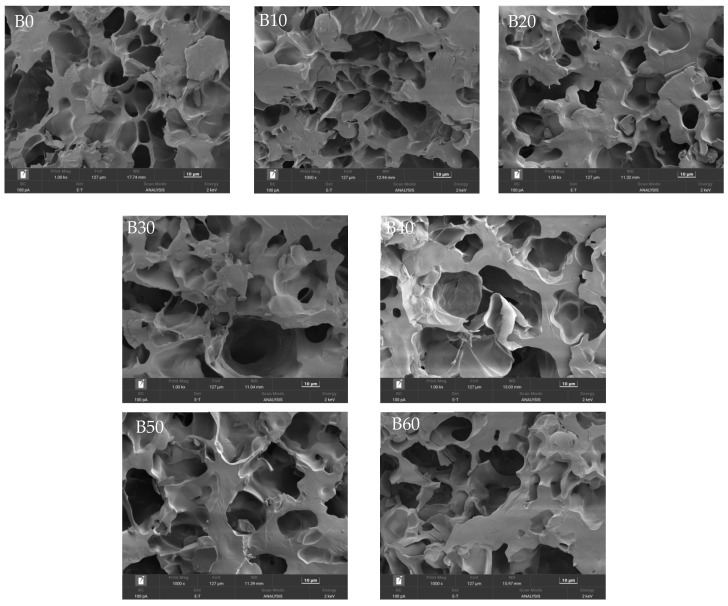
Cross-sectional microstructure of fresh wet tuber-based vermicelli at different immersion times. Images were captured at a magnification of 1000×; the scale bar represents 10 μm.

As shown in Figure 3, the cross-sections of fresh wet tuber-based vermicelli at different immersion times exhibited a porous gel network structure. With increased immersion time and the associated rise in moisture content, the network structure became looser, and the pore size gradually enlarged. This structural change led to a reduction in the hardness of the starch gel [19], which is consistent with the texture profile analysis presented earlier. However, when the immersion time reached 50 min, the internal pore structure began to disintegrate due to the excessive moisture. Therefore, an immersion time of 30–40 min, corresponding to a moisture content of 62–63%, is optimal to achieve desirable hardness while preserving the structural integrity of the fresh wet tuber-based vermicelli.

#### 3.1.6. Correlation Analysis

**Figure 4:**
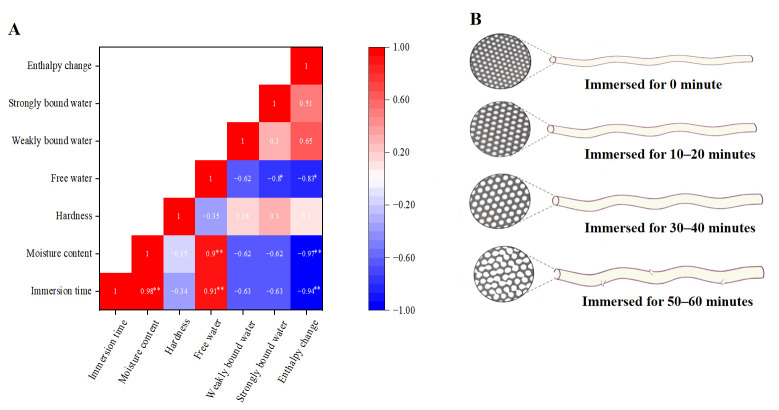
(**A**) Correlation analysis of the immersion time, moisture content, hardness, thermal enthalpy value and water in different states. Asterisks: * *p* < 0.05, ** *p* < 0.01. (**B**) Schematic of the variation in fresh wet tuber-based vermicelli with immersion time.

As shown in Figure 4A, a highly significant positive correlation (*p* < 0.01) was observed between the immersion time of fresh wet tuber-based vermicelli and both moisture content and free water proportion, with Pearson correlation coefficients exceeding 0.90. This indicates that longer immersion times significantly promote increases in both overall moisture and free water content. Conversely, a highly significant negative correlation (*p* < 0.01) was found both between immersion time and enthalpy change (−0.94), and between moisture content and enthalpy change (−0.97). This suggests that increased immersion time and the consequent higher moisture content significantly reduce the enthalpy change. Furthermore, immersion time showed negative correlations with hardness, weakly bound water, and strongly bound water, with coefficients of −0.34, −0.63, and −0.63, respectively. This implies that longer immersion times inhibit increases in hardness and the proportions of both weakly and strongly bound water. Regarding hardness, its Pearson correlation coefficients with free water, weakly bound water, and strongly bound water were −0.35, 0.18, and 0.30, respectively. This demonstrates that an increase in free water content effectively reduces the hardness of the vermicelli. Figure 4B depicts a schematic diagram illustrating the changes in fresh wet tuber-based vermicelli subjected to different immersion times. The longer the immersion time, the looser the gel network and the larger the pore size. At 30–40 min of immersion, the starch gel exhibited uniform and intact pore structures.

### 3.2. Effect of Immersion Time on the Physicochemical Properties of Fresh Wet Tuber-Based During Storage

#### 3.2.1. Moisture Distribution Analysis During Storage

As shown in Table 2, although the fresh wet tuber-based vermicelli samples had different initial moisture contents, the overall patterns of water migration during storage showed similarities. Over the first 21 days, a general trend was observed where the proportion of weakly bound water tended to decrease, while that of strongly bound water increased, despite some day-to-day fluctuations in the data. The initial increase in strongly bound water may be attributed to amylopectin retrogradation and gel network reinforcement [20]. Subsequently, with further storage, the content of strongly bound water showed an overall decreasing trend, which may be due to starch aggregation enhancing water mobility [20]. By 35 days of storage, the water distribution in vermicelli immersed for 30–40 min appeared to reach a plateau, with values fluctuating within a narrower range compared to earlier time points. In contrast, vermicelli immersed for 50–60 min (with a moisture content of 64–65%) exhibited a significantly higher free water content compared to other groups (*p* < 0.05). This is likely a result of structural disruption in the gel network pores, which impaired water retention capacity and facilitated the release of free water from the matrix. The increase in free water content during storage may compromise product stability and raise potential food safety concerns.

#### 3.2.2. Hardness Analysis During Storage

As shown in Table 3, the hardness of fresh wet tuber-based vermicelli increased progressively with extended storage time. This can be attributed to starch retrogradation, which induces realignment of starch molecules within the vermicelli and promotes crystalline formation, thereby reinforcing the structural integrity and leading to a continuous increase in hardness [21]. Furthermore, as indicated by the data, the hardness remained largely stable after 21 days of storage, suggesting that retrogradation was essentially complete by this time. After 49 days of storage, the final hardness values reached 6334 g for sample B0 (an increase of 4110 g) and 3144 g for sample B60 (an increase of 2115 g). The smaller increase in hardness observed in B60 was primarily due to its higher moisture content. In such high-moisture systems, water acts as a diluent, increasing the distance between starch molecules and reducing the probability of molecular interactions required for retrogradation. This impedes the reorganization of starch chains into crystalline structures and subsequently inhibits hardening [11]. These results demonstrate that higher moisture content can effectively retard the hardening of fresh wet tuber-based vermicelli during storage. However, this storage-induced structural hardening fundamentally alters the gel’s properties, affecting its performance upon subsequent reheating, as discussed in Section 3.3.2.

#### 3.2.3. Thermodynamic Properties Analysis During Storage

As shown in Table 4, the enthalpy change (ΔH) of fresh wet tuber-based vermicelli with different immersion times increased significantly (*p* < 0.05) during storage, showing a notable rise after 35 days and reaching a maximum value at day 49. This indicates that the extent of starch retrogradation progressed with prolonged storage time. Furthermore, longer immersion times resulted in lower final ΔH values at day 49. Specifically, the ΔH value was 4.96 J/g for sample B0 and 4.38 J/g for sample B60. This reduction can be attributed to higher moisture content, which decreased starch molecular concentration and thus limited the formation of crystalline structures. Additionally, the increased moisture enhanced the degree of gelatinization, which also contributed to the decrease in enthalpy value. These findings align with the trend observed in the initial enthalpy changes among vermicelli samples subjected to different immersion times.

#### 3.2.4. Microstructure Analysis During Storage

**Figure 5:**
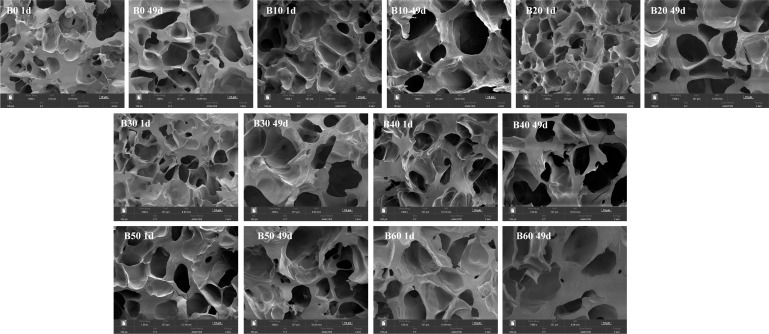
Microstructure of the cross-section of fresh wet tuber-based vermicelli at different immersion times during storage. Images were captured at a magnification of 1000×; the scale bar represents 10 μm.

As shown in Figure 5, the pore diameter of samples stored for 49 days was significantly larger than that of samples stored for 1 day. This phenomenon is attributed to starch retrogradation. Molecular recrystallization during retrogradation expels water from the crystalline regions into the amorphous gel matrix. This reduces the network’s water-holding capacity. During subsequent freeze-drying, the accumulated water sublimates, resulting in the observed larger pores in the microstructure. A higher degree of recrystallization reduces the water retention capacity of the gel network, leading to increased water mobility. Consequently, the gel network exhibits larger pores. Specifically, unimmersed fresh wet tuber-based vermicelli exhibited a small and dense starch gel structure. Samples immersed for 30–40 min (moisture content: 62–63%) maintained uniform pore size and a well-preserved gel network structure even after 49 days of storage. In contrast, vermicelli immersed for 50–60 min (moisture content: 64–65%) showed significantly enlarged pores and more severe structural disruption in the gel network after storage. Combined with the hardness changes during storage, these results indicate that moderate immersion (30–40 min) promotes the formation and stability of the starch gel structure. Although prolonged immersion (50–60 min) resulted in lower hardness throughout storage, it also led to an unstable gel network that was more susceptible to structural damage.

### 3.3. Effect of Immersion Time on the Reheating Quality of Fresh Wet Tuber-Based Vermicelli During Storage

#### 3.3.1. Water Absorption Rate Analysis

As shown in Table 5, following one day of storage, the water absorption rate of the reheated fresh wet tuber-based vermicelli ranged from 8% to 13%. Furthermore, with prolonged immersion time, the water absorption capacity decreased progressively, with the rate declining from 12.74% to 8.29%. In addition, a significant increase in water absorption rate was observed by day 7 of storage compared to day 1 (*p* < 0.05). This may be attributed to the short-term retrogradation of the vermicelli, during which the formation of a starch gel network enhanced water absorption upon reheating. Beyond 7 days of storage, the water absorption rates across all groups remained relatively stable.

#### 3.3.2. Hardness Analysis

It is important to distinguish between the hardness of non-reheated samples and that after reheating, as they reflect different structural states. The increase in hardness of non-reheated samples during storage (Table 3) is a direct indicator of starch retrogradation, which strengthens the gel network. However, this retrogradation also renders the network more rigid and brittle. Upon reheating in boiling water, this brittle structure undergoes uneven water absorption and partial disintegration, leading to a significant decrease in the overall hardness measured after reheating (Table 6). As shown in Table 6, with prolonged immersion time, the hardness of fresh wet tuber-based vermicelli stored for 1 day significantly decreased after reheating (*p* < 0.05). Furthermore, at any given immersion time, the hardness after storage and reheating was significantly lower (*p* < 0.05) than their unstored state. For example, sample B0 decreased from 2224 g (unstored state) to 719 g (stored 1 day and reheated), a reduction of approximately 68%. This may be attributed to starch recrystallization during storage, which reduced the stability of the starch gel network and consequently led to quality deterioration [22]. The vermicelli with longer immersion time (60 min) exhibited higher moisture content and weaker starch gel network stability, resulting in significantly reduced hardness and weaker cooking resistance after reheating. In contrast, samples with shorter immersion time (≤20 min) had lower moisture content, which promoted faster starch retrogradation during long-term storage. Consequently, these samples exhibited a more pronounced increase in hardness over the storage period compared to samples with longer immersion times (e.g., B60). It is hypothesized that these samples require more energy for re-gelatinization, necessitating extended reheating time and diminishing practical convenience.

## 4. Conclusions

During the 0–60 min immersion process, the moisture content of fresh wet tuber-based vermicelli increased significantly with prolonged immersion time. This higher moisture content diluted the starch molecules, reducing opportunities for intermolecular cross-linking and ordered polymer realignment. It also promoted starch gelatinization, thereby lowering the gelatinization enthalpy. Moreover, the increased moisture content elevated the proportion of free water and enlarged the pores in the gel network, which significantly reduced the hardness. An immersion time of 30–40 min was found to be optimal for achieving desirable hardness and structural integrity. During storage at 4 °C, the water distribution in vermicelli immersed for 30–40 min stabilized after 35 days. In contrast, vermicelli immersed for 50–60 min (moisture content: 64–65%) exhibited a significantly higher free water content and the lowest hardness value after stabilization, which is attributable to structural disruption and increased looseness. After reheating, the hardened crystalline structures formed via retrogradation were disrupted upon absorption of boiling water, leading to a consistent decrease in hardness across all groups over extended storage. Therefore, provided that the shelf life is ensured, controlling the immersion time to 30–40 min (corresponding to a moisture content of 62–63%) not only maintains adequate moisture and enhances gelatinization but also effectively mitigates hardness changes during storage and improves post-reheating quality. While this study focused on elucidating the physicochemical mechanisms, the established relationships between immersion time, moisture state, and textural properties provide a critical theoretical foundation for future sensory evaluation and product optimization aimed at aligning physicochemical stability with consumer acceptability. This study provides a theoretical basis for optimizing the storage quality and production efficiency of fresh wet tuber-based vermicelli.

## Figures and Tables

**Table 1:** Changes in transverse relaxation time of water in various states in fresh wet tuber-based vermicelli in different immersion times.

Samples	T_21_ (ms)	T_22_ (ms)	T_23_ (ms)	T_24_ (ms)
B0	0.26 ± 0.003 ^b^	1.21 ± 0.115 ^cd^	8.43 ± 0.834 ^c^	144.81 ± 0.005 ^d^
B10	0.33 ± 0.135 ^b^	-	9.34 ± 0.455 ^bc^	113.64 ± 5.575 ^e^
B20	0.63 ± 0.152 ^a^	-	9.34 ± 0.396 ^bc^	219.64 ± 0.005 ^c^
B30	0.20 ± 0.086 ^b^	1.48 ± 0.004 ^b^	9.60 ± 1.066 ^bc^	261.43 ± 12.827 ^b^
B40	0.14 ± 0.004 ^b^	1.39 ± 0.135 ^bc^	10.05 ± 1.480 ^bc^	257.96 ± 17.728 ^b^
B50	0.30 ± 0.127 ^b^	1.12 ± 0.004 ^d^	11.12 ± 1.091 ^b^	289.94 ± 0.005 ^a^
B60	0.42 ± 0.136 ^ab^	1.96 ± 0.004 ^a^	13.70 ± 1.344 ^a^	300.04 ± 14.274 ^a^

Different letters in the same column indicate significant differences (*p* < 0.05). The symbol “-” indicates that the corresponding value was not detected.

**Table 2:** Changes in the relative proportions of water populations in fresh wet tuber-based vermicelli during storage.

Storage Time (d)	Samples	The Relative Content of Free Water (%)	The Relative Content of Weakly Bound Water (%)	The Relative Content of Strongly Bound Water (%)
1	B0	0.51 ± 0.040 ^BCa^	89.05 ± 1.040 ^Bd^	10.45 ± 1.002 ^Ca^
	B10	0.16 ± 0.021 ^Be^	96.37 ± 0.071 ^Ab^	3.48 ± 0.051 ^Ec^
	B20	0.05 ± 0.002 ^Cf^	97.70 ± 0.073 ^Aa^	2.25 ± 0.069 ^Dc^
	B30	0.03 ± 0.012 ^Df^	97.21 ± 0.089 ^Aab^	2.76 ± 0.078 ^Dc^
	B40	0.37 ± 0.031 ^Cc^	92.34 ± 0.231 ^Ac^	7.30 ± 0.260 ^Cb^
	B50	0.22 ± 0.011 ^Dd^	89.50 ± 0.819 ^Ad^	10.28 ± 0.812 ^Aa^
	B60	0.42 ± 0.020 ^Cb^	93.37 ± 0.431 ^Ac^	6.21 ± 0.410 ^Db^
3	B0	0.90 ± 0.003 ^Bc^	84.88 ± 1.062 ^Dc^	14.22 ± 1.060 ^Ac^
	B10	1.10 ± 0.102 ^Ab^	79.34 ± 0.071 ^Ed^	19.57 ± 0.179 ^Aa^
	B20	0.73 ± 0.011 ^Ac^	83.37 ± 0.870 ^Dc^	15.91 ± 0.892 ^Abc^
	B30	0.89 ± 0.112 ^Bc^	96.53 ± 0.190 ^Aa^	2.58 ± 0.081 ^Df^
	B40	1.86 ± 0.072 ^Aa^	81.06 ± 1.590 ^Cd^	17.08 ± 1.518 ^Ab^
	B50	1.80 ± 0.100 ^Ba^	89.74 ± 0.412 ^Ab^	8.46 ± 0.311 ^ABe^
	B60	0.50 ± 0.043 ^Cd^	88.95 ± 0.732 ^CDb^	10.55 ± 0.692 ^Bd^
7	B0	0.24 ± 0.020 ^Cc^	86.66 ± 0.662 ^CDc^	13.10 ± 0.683 ^ABb^
	B10	0.28 ± 0.003 ^Bbc^	83.97 ± 0.751 ^Dd^	15.75 ± 0.759 ^Ba^
	B20	0.64 ± 0.012 ^Aa^	83.56 ± 0.821 ^Dd^	15.81 ± 0.810 ^Aa^
	B30	0.27 ± 0.010 ^CDbc^	88.06 ± 0.370 ^Cb^	11.67 ± 0.359 ^Ac^
	B40	0.59 ± 0.111 ^Ca^	87.35 ± 0.079 ^Bbc^	12.07 ± 0.189 ^Bbc^
	B50	0.64 ± 0.051 ^Ca^	91.05 ± 0.302 ^Aa^	8.31 ± 0.351 ^ABd^
	B60	0.37 ± 0.020 ^Cb^	87.38 ± 0.492 ^Ebc^	12.26 ± 0.471 ^Abc^
21	B0	0.55 ± 0.031 ^BCe^	87.63 ± 0.860 ^BCb^	11.83 ± 0.890 ^BCab^
	B10	0.25 ± 0.012 ^Bf^	86.82 ± 0.631 ^Cb^	12.94 ± 0.619 ^Ca^
	B20	0.14 ± 0.011 ^Cg^	88.34 ± 0.910 ^Cb^	11.53 ± 0.901 ^Bab^
	B30	1.79 ± 0.031 ^Aa^	88.47 ± 0.442 ^Cb^	9.73 ± 0.471 ^Bcd^
	B40	1.01 ± 0.010 ^Bc^	87.87 ± 0.572 ^Bb^	11.12 ± 0.583 ^Bbc^
	B50	0.78 ± 0.082 ^Cc^	90.30 ± 0.649 ^Aa^	8.92 ± 0.572 ^Ad^
	B60	1.29 ± 0.081 ^Bb^	87.89 ± 0.810 ^Db^	10.82 ± 0.731 ^Bbc^
35	B0	0.65 ± 0.040 ^Ad^	93.42 ± 0.377 ^Ab^	5.93 ± 0.389 ^Da^
	B10	0.91 ± 0.061 ^Ac^	93.21 ± 0.223 ^Ba^	5.89 ± 0.219 ^Da^
	B20	0.51 ± 0.041 ^ABe^	93.60 ± 0.552 ^Ba^	5.89 ± 0.578 ^Ca^
	B30	0.48 ± 0.052 ^Be^	93.34 ± 0.310 ^Ba^	6.18 ± 0.291 ^Ca^
	B40	0.42 ± 0.013 ^Ce^	93.36 ± 0.441 ^Aa^	6.22 ± 0.439 ^Ca^
	B50	2.77 ± 0.201 ^Ab^	91.11 ± 0.449 ^Ac^	6.12 ± 0.541 ^Ba^
	B60	3.22 ± 0.222 ^Aa^	90.64 ± 0.110 ^Bc^	6.14 ± 0.260 ^Da^
49	B0	0.46 ± 0.041 ^BCc^	92.66 ± 0.181 ^Ab^	6.87 ± 0.129 ^Dbc^
	B10	0.28 ± 0.020 ^Bd^	93.46 ± 0.629 ^Bb^	6.26 ± 0.630 ^Dc^
	B20	0.29 ± 0.030 ^BCcd^	94.64 ± 0.519 ^Ba^	5.07 ± 0.519 ^Cd^
	B30	0.45 ± 0.029 ^Ccd^	92.93 ± 0.640 ^Bb^	6.62 ± 0.610 ^Cc^
	B40	0.29 ± 0.031 ^Ccd^	93.48 ± 0.190 ^Ab^	6.24 ± 0.210 ^Cc^
	B50	1.77 ± 0.102 ^Bb^	90.20 ± 0.678 ^Ac^	8.03 ± 0.651 ^ABa^
	B60	2.62 ± 0.220 ^Aa^	89.80 ± 0.349 ^BCc^	7.58 ± 0.520 ^Cab^

For comparisons among samples (different immersion times) within the same storage day, means followed by different lowercase letters are significantly different (*p* < 0.05). For comparisons across storage days within the same sample, means followed by different uppercase letters are significantly different (*p* < 0.05).

**Table 3:** Changes in hardness (g) of fresh wet tuber-based vermicelli during storage.

Storage Time (d)	B0	B10	B20	B30	B40	B50	B60
1	2224.56 ± 81.39 ^Ca^	2215.37 ± 89.28 ^Da^	1700.20 ± 48.29 ^Cb^	1298.29 ± 29.13 ^Cc^	1111.35 ± 64.79 ^Dd^	1060.48 ± 34.05 ^Cd^	1029.44 ± 59.30 ^Bb^
7	3912.74 ± 162.60 ^Ba^	3938.44 ± 264.68 ^Ca^	3190.87 ± 209.21 ^Bb^	2901.69 ± 231.48 ^Bb^	2956.36 ± 154.80 ^Cb^	2653.03 ± 112.35 ^Bc^	2098.72 ± 207.01 ^Bd^
21	6528.45 ± 281.68 ^Aa^	4906.39 ± 304.06 ^Bb^	4067.33 ± 191.47 ^Ac^	4134.39 ± 149.32 ^Ac^	3728.45 ± 322.50 ^Bc^	3799.09 ± 203.11 ^Ac^	3158.56 ± 230.07 ^Ad^
35	6303.35 ± 194.10 ^Aa^	5693.11 ± 290.20 ^Ab^	4303.96 ± 173.32 ^Ac^	4364.37 ± 290.65 ^Ac^	4328.79 ± 224.36 ^Ac^	3601.79 ± 175.20 ^Ad^	3161.48 ± 143.30 ^Ae^
49	6334.49 ± 111.38 ^Aa^	5530.28 ± 141.35 ^Ab^	4473.54 ± 129.07 ^Ac^	4334.40 ± 112.30 ^Ac^	4421.23 ± 164.60 ^Ac^	3486.39 ± 136.08 ^Ad^	3144.65 ± 203.04 ^Ad^

For comparisons among samples (different immersion times) within the same storage day, means followed by different lowercase letters are significantly different (*p* < 0.05). For comparisons across storage days within the same sample, means followed by different uppercase letters are significantly different (*p* < 0.05).

**Table 4:** Changes in thermal enthalpy value (ΔH, J/g) of fresh wet tuber-based vermicelli during storage.

Storage Time (d)	B0	B10	B20	B30	B40	B50	B60
1	0.92 ± 0.051 ^Ca^	0.92 ± 0.042 ^Ca^	0.82 ± 0.010 ^Cb^	0.69 ± 0.020 ^Dc^	0.69 ± 0.010 ^Cc^	0.60 ± 0.079 ^Dd^	0.40 ± 0.030 ^De^
7	1.00 ± 0.069 ^Ca^	0.58 ± 0.019 ^Dc^	0.92 ± 0.082 ^Cab^	0.79 ± 0.031 ^CDb^	0.78 ± 0.003 ^Cbc^	0.72 ± 0.003 ^Dbc^	0.76 ± 0.032 ^Cbc^
21	0.90 ± 0.078 ^Cab^	0.70 ± 0.010 ^CDc^	0.81 ± 0.051 ^Cbc^	0.94 ± 0.011 ^Cab^	0.97 ± 0.031 ^Cab^	1.00 ± 0.071 ^Ca^	0.86 ± 0.031 ^Cabc^
35	3.95 ± 0.220 ^Ba^	3.35 ± 0.220 ^Bab^	3.31 ± 0.210 ^Bab^	3.23 ± 0.121 ^Bab^	3.27 ± 0.282 ^Bab^	3.01 ± 0.181 ^Bb^	2.65 ± 0.230 ^Bb^
49	4.96 ± 0.251 ^Aa^	4.85 ± 0.101 ^Aab^	4.90 ± 0.121 ^Aa^	4.86 ± 0.163 ^Aab^	4.83 ± 0.072 ^Aab^	4.55 ± 0.141 ^Aab^	4.38 ± 0.091 ^Ab^

For comparisons among samples (different immersion times) within the same storage day, means followed by different lowercase letters are significantly different (*p* < 0.05). For comparisons across storage days within the same sample, means followed by different uppercase letters are significantly different (*p* < 0.05).

**Table 5:** Changes in water absorption rate (%) of fresh wet tuber-based vermicelli during storage.

Storage Time (d)	B0	B10	B20	B30	B40	B50	B60
1	12.74 ± 0.75 ^Ca^	10.72 ± 0.18 ^Cb^	10.64 ± 0.92 ^Cb^	10.14 ± 0.07 ^Db^	9.96 ± 0.81 ^Dbc^	8.74 ± 0.25 ^Cc^	8.29 ± 0.42 ^Dc^
7	18.95 ± 0.55 ^Aa^	17.10 ± 0.83 ^ABab^	16.03 ± 0.56 ^Ab^	16.58 ± 0.30 ^ABb^	17.21 ± 1.73 ^ABab^	14.54 ± 0.28 ^Bd^	15.48 ± 0.15 ^Ac^
21	18.60 ± 0.56 ^Aa^	18.30 ± 0.85 ^Aa^	16.48 ± 0.59 ^Abc^	17.56 ± 0.07 ^Aab^	17.70 ± 0.28 ^Bab^	14.87 ± 1.35 ^ABc^	15.62 ± 0.86 ^ABc^
35	18.98 ± 0.56 ^Aa^	18.11 ± 0.56 ^Aa^	14.28 ± 1.12 ^Bbc^	13.48 ± 0.78 ^Cc^	18.70 ± 0.11 ^Aa^	15.70 ± 0.22 ^Ab^	13.79 ± 0.12 ^Cc^
49	16.67 ± 0.22 ^Ba^	16.55 ± 0.13 ^Ba^	16.50 ± 0.04 ^Aa^	16.19 ± 0.23 ^Ba^	15.98 ± 0.73 ^Cab^	14.30 ± 0.42 ^Bb^	14.29 ± 0.47 ^Bb^

For comparisons among samples (different immersion times) within the same storage day, means followed by different lowercase letters are significantly different (*p* < 0.05). For comparisons across storage days within the same sample, means followed by different uppercase letters are significantly different (*p* < 0.05).

**Table 6:** Changes in hardness (g) of fresh wet tuber-based vermicelli after reheating during storage.

Storage Time (d)	B0	B10	B20	B30	B40	B50	B60
1	719.09 ± 28.10 ^Aa^	645.33 ± 29.48 ^Ab^	523.48 ± 42.30 ^Ac^	388.97 ± 36.10 ^Ad^	384.33 ± 22.01 ^Ad^	241.29 ± 12.01 ^Ae^	232.11 ± 6.28 ^Ae^
7	343.11 ± 11.40 ^Ba^	258.60 ± 16.73 ^BCb^	231.01 ± 17.67 ^Cbc^	208.38 ± 7.06 ^BCc^	199.46 ± 7.11 ^Bc^	204.67 ± 12.30 ^Bc^	162.20 ± 7.03 ^CDd^
21	256.80 ± 13.22 ^Ca^	217.30 ± 11.02 ^Db^	198.04 ± 11.00 ^Dbc^	204.40 ± 11.97 ^BCb^	176.05 ± 9.10 ^Ccd^	176.29 ± 10.01 ^Ccd^	172.30 ± 4.66 ^Cd^
35	269.33 ± 12.69 ^Ca^	251.30 ± 8.12 ^Ca^	263.20 ± 9.03 ^Ba^	191.65 ± 7.03 ^Cb^	182.93 ± 6.01 ^Cb^	162.20 ± 4.32 ^Cc^	189.79 ± 10.08 ^Bb^
49	266.30 ± 5.70 ^Ca^	256.39 ± 4.31 ^Ca^	222.83 ± 8.02 ^Cb^	184.83 ± 9.01 ^Dc^	190.29 ± 10.01 ^BCc^	161.01 ± 8.05 ^Cd^	155.36 ± 2.01 ^Dd^

For comparisons among samples (different immersion times) within the same storage day, means followed by different lowercase letters are significantly different (*p* < 0.05). For comparisons across storage days within the same sample, means followed by different uppercase letters are significantly different (*p* < 0.05).

## Data Availability

The original contributions presented in the study are included in the article, further inquiries can be directed to the corresponding author.

## References

[1] Feng Y.-Y., Mu T.-H., Zhang M., Ma M.-M. (2020). Effects of different polysaccharides and proteins on dough rheological properties, texture, structure and in vitro starch digestibility of wet sweet potato vermicelli. Int. J. Biol. Macromol..

[2] Feng Y.-Y., Mu T.-H., Zhang M., Ma M.-M. (2020). Effects of ionic polysaccharides and egg white protein complex formulations on dough rheological properties, structure formation and in vitro starch digestibility of wet sweet potato vermicelli. Int. J. Biol. Macromol..

[3] Liu T., Men Z., Lai C., Lian X. (2025). Preparation and Mechanism Analysis of Boiling Resistance of the Fresh Alum-Free Sweet Potato Vermicelli Containing Gliadin Fractions. Foods.

[4] Yi C., Zhu H., Tong L., Zhou S., Yang R., Niu M. (2019). Volatile profiles of fresh rice noodles fermented with pure and mixed cultures. Food Res. Int..

[5] Yang S., Dhital S., Shan C.-S., Zhang M.-N., Chen Z.-G. (2021). Ordered structural changes of retrograded starch gel over long-term storage in wet starch noodles. Carbohydr. Polym..

[6] Qiu L., Zhang M., Tang J., Adhikari B., Cao P. (2019). Innovative technologies for producing and preserving intermediate moisture foods: A review. Food Res. Int.

[7] Jin X., Cheng L., Hong Y., Li Z., Li C., Ban X., Gu Z. (2023). Effect of heat-moisture treatment (HMT) on thermal stability of starch gel and the surface adhesiveness of vermicelli. Int. J. Biol. Macromol..

[8] Liu L., Shi Z., Wang X., Ren T., Ma Z., Li X., Xu B., Hu X. (2021). Interpreting the correlation between repeated sheeting process and wheat noodle qualities: From water molecules movement perspective. LWT.

[9] Pouplin M., Redl A., Gontard N. (1999). Glass Transition of Wheat Gluten Plasticized with Water, Glycerol, or Sorbitol. J. Agric. Food Chem..

[10] Slade L., Levine H., Reid D.S. (1991). Beyond water activity: Recent advances based on an alternative approach to the assessment of food quality and safety. Crit. Rev. Food Sci. Nutr..

[11] Jane K.A., Inamdar N.N., Kotagale N.R. (2025). Effect of gelatinizing temperature and moisture on the retrogradation of coix starch. Int. J. Biol. Macromol..

[12] Zhang Y., Zhang J., Wang Z., Fan L., Chen Y. (2024). Effect of Rice Protein on the Gelatinization and Retrogradation of Rice Starch with Different Moisture Content. Foods.

[13] (2016). National Food Safety Standard—Determination of Moisture in Foods.

[14] Huang R., Huang K., Song H., Li S., Guan X. (2025). Evaluation of extruded quinoa flour on dough rheology and white salted noodles quality. J. Food Sci..

[15] Hu Y., Li C., Tan Y., McClements D.J., Wang L. (2022). Insight of rheology, water distribution and in vitro digestive behavior of starch based-emulsion gel: Impact of potato starch concentration. Food Hydrocoll..

[16] Yan Y., Jia M., Zhou Z., Xiao S., Lin P., Wang Y., Fu Y., Wang X. (2025). Effect of ultrasonic treatment on the physicochemical properties of buckwheat starch: Based on the ultrasonic power and moisture content. Ultrason. Sonochem..

[17] Wang M., Chen J., Chen S., Ye X., Liu D. (2021). Inhibition effect of three common proanthocyanidins from grape seeds, peanut skins and pine barks on maize starch retrogradation. Carbohydr. Polym..

[18] Li E., Cao P., Cao W., Li C. (2022). Relations between starch fine molecular structures with gelatinization property under different moisture content. Carbohydr. Polym..

[19] Yu W., Yu Y., Li J., Liang H., Li Y., Li B. (2025). Effects of deacetylated konjac glucomannan on the retrogradation properties of pea, mung bean and potato starches during the storage. Int. J. Biol. Macromol..

[20] Chen L., Tian Y., Tong Q., Zhang Z., Jin Z. (2017). Effect of pullulan on the water distribution, microstructure and textural properties of rice starch gels during cold storage. Food Chem..

[21] Zhai Y., Li X., Bai Y., Jin Z., Svensson B. (2022). Maltogenic α-amylase hydrolysis of wheat starch granules: Mechanism and relation to starch retrogradation. Food Hydrocoll..

[22] Gaikwad S., Arya S.S. (2018). Influence of frozen storage on quality of multigrain dough, par baked and ready to eat thalipeeth with additives. LWT.

